# Molecular Dosimetry of DNA Adducts in Rats Exposed to Vinyl Acetate Monomer

**DOI:** 10.1093/toxsci/kfab140

**Published:** 2021-12-14

**Authors:** Yun-Chung Hsiao, Chih-Wei Liu, Gary Hoffman, Caroline Fang, Kun Lu

**Affiliations:** Department of Environmental Sciences and Engineering, University of North Carolina at Chapel Hill, Chapel Hill, North Carolina 27599, USA; Department of Environmental Sciences and Engineering, University of North Carolina at Chapel Hill, Chapel Hill, North Carolina 27599, USA; Covance CRS, LLC, Somerset, New Jersey 08873, USA; Department of Environmental Sciences and Engineering, University of North Carolina at Chapel Hill, Chapel Hill, North Carolina 27599, USA; Department of Environmental Sciences and Engineering, University of North Carolina at Chapel Hill, Chapel Hill, North Carolina 27599, USA

**Keywords:** cancer, vinyl acetate monomer, DNA adduct, risk assessment, LC-MS/MS, mass spectrometry, molecular dosimetry

## Abstract

Vinyl acetate monomer (VAM) is heavily used to synthesize polymers. Previous studies have shown that inhaled VAM, being metabolized to acetaldehyde, may form DNA adducts including N^2^-ethylidene-deoxyguanosine (N^2^-EtD-dG), which may subsequently cause mutations and contribute to its carcinogenesis. Currently, there is little knowledge on the molecular dosimetry between VAM exposure and DNA adducts under dosages relevant to human exposure. In this study, 0.02, 0.1, 1, 10, 50, 200, and 600 ppm VAM were exposed to rats by inhalation for 14 days (6 h/day). The use of [^13^C_2_]-VAM allows unambiguous differentiation and quantification of the exogenous and endogenous N^2^-EtD-dG by highly sensitive LC-MS/MS. Our data indicate that VAM-induced exogenous DNA adducts were formed in a non-linear manner. Exogenous DNA adducts were only detected in the nasal epithelium of rats exposed to 10, 50, 200, and 600 ppm VAM, whereas endogenous adducts were found in all nasal and other tissues analyzed. In addition, ratios of exogenous/endogenous DNA adducts were less than 1 with the dose up to 50 ppm, indicating that endogenous DNA adducts are predominant at low VAM concentrations. Moreover, differential dose-response in terms of exogenous DNA adduct formation were observed between nasal respiratory and olfactory epithelium. Furthermore, the lack of exogenous DNA adducts in distant tissues, including peripheral blood mononuclear cells, liver, brain, and bone marrow, indicates that VAM and/or its metabolite do not distribute systemically to cause DNA damage in distant tissues. Together, these results provided new molecular dosimetry to improve science-based cancer risk assessments of VAM.

##  

Vinyl acetate monomer (VAM) is an important industrial intermediate, and synthesized polymers have versatile applications including adhesives, coatings, paints, and other end-products. However, whether VAM is carcinogenic in human remains debatable. The International Agency for Research on Cancer (IARC) listed VAM as group 2B, which indicates possibly carcinogenic but lack of evidence in animal and human studies (IARC, 1987). Human exposure to VAM can happen for workers involved in VAM production and polymerization, and for general population when polymerized end-products release residual VAM (Albertini, 2013).

Previous *in vitro* and *in vivo* toxicological studies have suggested the potential mechanisms underlying VAM carcinogenicity (Albertini, 2013; Bogdanffy, 1999; Bogdanffy *et al.*, 1994; Kuykendall *et al.*, 1993; Lambert *et al.*, 1985). Figure  1A illustrates how the key event in cancer development, that is, formation of DNA adducts, can occur after exposure to VAM. Absorbed VAM is first metabolized into acetaldehyde and acetic acid by the carboxylesterase (CE). VAM-derived acetaldehyde, if not deactivated by the aldehyde hydrogenase (ALDH) family, attacks nucleotides and forms DNA adducts including N^2^-ethylidene-deoxyguanosine (N^2^-EtD-dG) and 1, N^2^-propano-dG, with N^2^-EtD-dG being the primary DNA adducts as shown in our previous study (Liu *et al.*, 2021). For convenience, DNA adducts, such as N^2^-EtD-dG, induced by inhaled VAM rather than from endogenous acetaldehyde are referred as “exogenous adducts” in this study.

**Figure 1.**
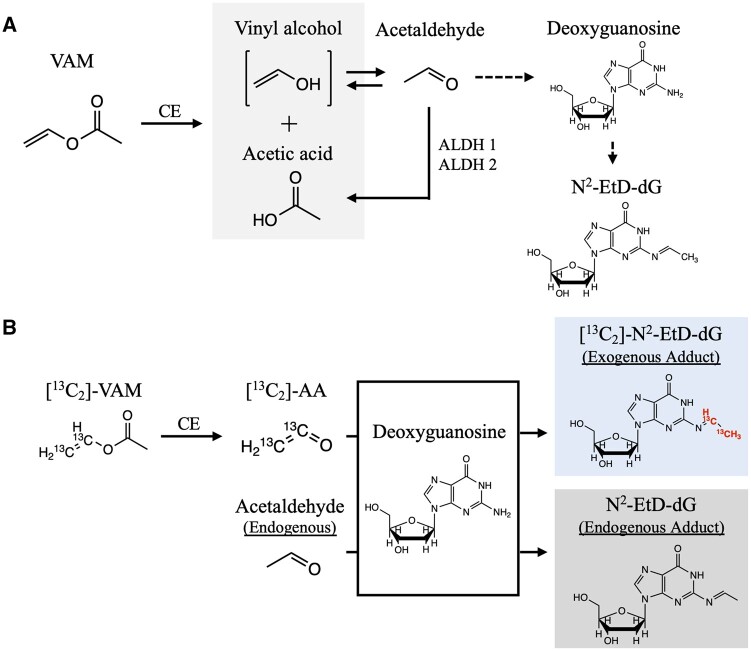
A, DNA adduct formation by exposure to VAM and B, differentiation of endogenous N^2^-EtD-dG and exogenous [^13^C_2_]-N^2^-EtD-dG by using [^13^C_2_]-VAM for exposure. Note: N^2^-EtD-dG is not stable and requires chemical reduction into stable N^2^-ethyl-dG (N^2^-Et-dG) for detection. VAM, vinyl acetate monomer; CE, carboxylesterase; ALDH, aldehyde dehydrogenase; N^2^-EtD-dG, N^2^-ethylidene-deoxyguanosine; AA, acetaldehyde.

DNA adducts, if not repaired efficiently and correctly, may lead to mutations, contributing to cancer development (Chatterjee and Walker, 2017). Thus, the formation of DNA adducts serves as a key event in carcinogenesis. DNA adducts have been frequently used as biomarkers of exposure to evaluate the cancer risk of chemicals (Beland *et al.*, 2005; Rundle, 2006). Thorough review on genotoxicity assessments related to VAM has been previously published (Albertini, 2013). DNA damage such as adduct formation and DNA-DNA crosslink have been reported for both VAM and its metabolite acetaldehyde in *in vitro* bioassays (Budinsky *et al.*, 2013; Jantunen *et al.*, 1986). *In vivo* studies have shown that nasal tumors were induced by VAM in rodents at high doses (Bogdanffy *et al.*, 1994; Owen, 1988). However, science-based toxicological assessment of VAM still suffers from the lack of high quality dose-response data. Molecular dosimetry between VAM exposure and corresponding DNA adducts remains unclear. One of the challenges in assessing VAM carcinogenicity is the existence of endogenous acetaldehyde. Acetaldehyde from VAM metabolism and endogenous physiological processes can both result in formation of N^2^-EtD-dG, which is undistinguishable in conventional assays. We have been developing methods by introducing stable isotope labeled chemicals for exposure to address this long-standing issue of the availability to reliable data in risk assessment of compounds with both endogenous and exogenous sources, such as VAM. As presented in this study, exogenous and endogenous N^2^-EtD-dG can be differentiated by mass spectrometry when stable isotope labeled [^13^C_2_]-VAM is used for exposure, which is illustrated in Figure  1B.

In our recent study, we have demonstrated the formation of exogenous N^2^-Et-dG in respiratory and olfactory epithelia of rat exposed to high doses of [^13^C_2_]-VAM (50, 200, and 400 ppm) for 6 h (Liu *et al.*, 2021). However, the molecular dosimetry of DNA adducts over a wide dose range critical for risk assessment of VAM remains unknown. In addition, our previous study used 1-day exposure (6 h) (Liu *et al.*, 2021), whereas DNA adducts would approach the steady state from repeated exposure. Therefore, the objective of this study was to establish the molecular dosimetry of DNA adducts in rats exposed to VAM for 14 days (6 h/day). Specifically, 0.02, 0.1, 1, 10, 50, 200, and 600 ppm VAM were exposed to rats for 6 h/day for 14 consecutive days. The doses we used ranged from highly human relevant low doses to the high concentrations that induced nasal tumors in rodent cancer bioassays. Proximal tissues including respiratory and olfactory epithelia in nasal cavity, and distant tissues including peripheral blood mononuclear cells (PBMCs), liver, brain, and bone marrow were all collected and analyzed for endogenous and exogenous DNA adducts by highly sensitive nano-LC-MS/MS methods.

## MATERIALS AND METHODS

###  

####  

##### Chemicals and materials

All reagents and chemicals, unless otherwise specified, were obtained from Sigma-Aldrich (St Louis, Missouri). High-performance liquid chromatography (HPLC) or Optimal LC-MS grade water, methanol, acetonitrile, and 2-propanol were purchased from Thermo Fisher Scientific (Rockford, Illinois). NucleoBond AXG 20 and AXG 100 anion-exchange columns and corresponding buffer kits were purchased from Macherey-Nagel (Bethlehem, Pennsylvania). Proteinase K was purchased from VWR International LLC (Atlanta, Georgia); stainless steel beads (5 mm) were from QIAGEN (Germantown, Maryland); and Nanosep centrifugal devices 3K were obtained from Pall Life Sciences (Port Washington, New York).

##### Rat exposure to VAM

Test atmospheres of VAM were generated by vaporizing [^13^C_2_]-VAM (CAS No. 106139-40-6, P/N: 493481, Batch number: MBBC9298) or [^12^C_2_]-VAM (unlabeled, CAS No. 108-05-4, P/N: V1503, Batch number: STBJ22968) obtained from Sigma-Aldrich at room temperature using acetone as the vehicle. The vapor concentration of samples drawn directly from the exposure system atmosphere were determined by a calibrated infrared (IR) spectrophotometer (for high doses groups only) and/or charcoal tubes and gas chromatography-flame ionization detector (GC-FID) (for low concentration groups). IR spectrophotometer monitored VAM concentrations in a real-time manner daily, and the details on GC-FID method are provided in Supplementary Table 1.

Animal use in this study was approved by the Institutional Animal Use and Care Committee of Covance and was conducted in accordance with the National Institutes of Health guidelines for the care and use of laboratory animals. Animals were housed in fully accredited American Association for Accreditation of Laboratory Animal Care facilities. Male Sprague-Dawley rats (8–10 weeks old, *n*: up to 20/group) were exposed to 0.02 (group 2), 0.1 (group 3), 1 (group 4), 10 (group 5), and 50 (group 6) ppm of stable isotope labeled [^13^C_2_]-VAM; or 200 (group 7) and 600 (group 8) ppm unlabeled [^12^C_2_]-VAM atmospheres for 14 days (6 h/day) using a single directed flow nose-only exposure system (manufactured by Covance, Huntingdon, UK). Rats exposed to acetone, the vehicle of VAM distribution, were prepared as controls (group 1). Nasal respiratory and olfactory epithelia and other distant tissues such as liver, brain, and bone marrow were collected from exposed rats for DNA adduct measurement. In addition, PBMCs were collected from rats within 1–2 h post-exposure to examine potential systemic distribution of [^13^C_2_]-VAM.

##### DNA extraction and enzymatic digestion

Extraction and digestion of DNA followed the experimental procedures described previously with minor modifications (Hsiao *et al.*, 2020; Liu *et al.*, 2021). In brief, collected rat tissues including nasal respiratory and olfactory epithelia, liver, brain, and bone marrow were first mechanically homogenized in G_2_ buffer (one of the corresponding buffers for NucleoBond columns) by stainless steel bead beating. Alternatively, isolated PBMCs were directly transferred to G_2_ buffer. These G_2_ buffer containing biological samples were extracted and purified for genomic DNA following the manufacturer’s instruction for NucleoBond AXG 20 or AXG 100. Those 2 cartridges contained identical packaging materials but with different loading capacity. The concentrations of purified DNA were measured with a Nanodrop One spectrophotometer (Thermo Fisher Scientific).

At least 10 µg DNA from each of the biological samples were used for further procedures. DNA was treated with NaBH_3_CN (50 mM) and sodium phosphate buffer (100 mM, pH 7.2) for 17 h at 37°C with gentle shaking to convert the N^2^-EtD-dG into N^2^-Et-dG form before digestion. The incubated solutions were then added with 200 µl of 50 mM sodium phosphate/20 mM MgCl_2_ (pH 7.2) buffer and 3.125 fmol of [^15^N_5_]-N^2^-Et-dG, [^13^$C_{5}^{15}$N_5_]-N^2^-ε-dG, and [^13^$C_{10}^{15}$N_5_]-1, N^2^-propano-dG that served as internal standards. Enzymatic digestion of DNA was done by the incubation with DNAse I (200 units), alkaline phosphatase (5 units), and phosphodiesterase (0.005 units) at 37°C for 1 h. The digests were filtered through a NanoSep 3 kDa filter at 8000 rpm for 40 min to remove enzymes before HPLC fractionation.

As stated, 10 µg of DNA was typically used for adduct analysis, however, we had to pool samples and used 120 µg of DNA for low does groups to increase the likelihood for exogenous adduct detection. Under such a situation, we have done extensive evaluations of potential contribution of natural isotopic abundances using standards and the same amount of digested DNA. The contribution of natural isotopic abundances is approximately 0.141% (mean). With over 100 consecutive runs to calculate the variation (standard deviation = 0.016%), we set the criteria of mean + 3 × standard deviation = 0.189% as the criteria to determine whether there is any real exogenous [^13^C_2_]-N^2^-Et-dG in samples using 120 µg of DNA.

##### HPLC fractionation and purification of DNA adducts

The hydrolyzed DNA solutions were injected onto an Agilent 1200 Series UV-HPLC that contained a fraction collector. The DNA adducts of interest were separated from the abundant common nucleosides and collected by reversed-phase liquid chromatography with an Atlantis C_18_ T3 column (150 × 4.6 mm, 3 µm, Waters) under 30°C column temperature. The mobile phases consisted of water with 10 mM ammonium acetate (A) and methanol (B) with the gradient set as the following: 5% B from 0 to 5 min, 5% to 15% B from 5 to 12 min, 15% to 22% B from 12 to 34 min, 22% to 80% B from 34 to 35 min, and a 5-min hold at 80% B for re-equilibration. N^2^-ε-dG and N^2^-Et-dG/1, N^2^-propano-dG fractions were collected between 20.5 to 22.5 min and 27.0 to 31.3 min, respectively. The signal area of UV absorption at wavelength 254 nm in each of the HPLC fractionation was used to calculate the amount of dG in each of the loaded samples by a calibration curve (Supplementary Figure 1).

##### Nano-LC−ESI-MS/MS analysis

An Ultimate 3000 RSLCnano system coupled to a Q Exactive HF Hybrid Quadrupole-Orbitrap mass spectrometer through an EASY-Spray ion source for nanoelectrospray ionization (Thermo Fisher Scientific) was used to perform nano-LC−ESI-MS/MS analysis for the detection of DNA adducts. Fractions collected from the HPLC system were dried in *vacuus* and reconstituted in 0.1% formic acid for analysis. The samples were first loaded into a C_18_ trapping cartridge (5 µm particle, 0.5 cm × 300 µm i.d., catalog no. 160454, Thermo Fisher Scientific) using 0.1% formic acid in water as loading solvent at 5 µl/min for 3 min. The trapped analytes were subsequently eluted to a PepMap C_18_ analytical column (2 µm particle, 25 cm × 75 µm i.d., catalog no. ES802A, Thermo Fisher Scientific) for separation prior to the detection of MS. The binary solvent system for mobile phase was consisted of water (A) and acetonitrile (B) both with 0.1% formic acid at a flow rate of 300 nl/min. The gradient was set as the following: 5% from 0 to 3 min (trapping time), 5% to 40% B from 3 to 16 min, 40% to 90% B from 16 to 16.1 min, 90% B from 16.1 to 26 min, 90% to 5% B from 26 to 26.1 min, and 5% B from 26.1 to 40 min as a final re-equilibration.

Targeted parallel reaction monitoring (PRM) mode was applied to collect LC-MS/MS raw data. For the detection of N^2^-Et-dG, an inclusion list composed of *m/z* 296.1353 (endogenous N^2^-Et-dG), *m/z* 298.1420 (exogenous [^13^C_2_]-N^2^-Et-dG), and *m/z* 301.1205 (internal standard [^15^N_5_]-N^2^-Et-dG) was used to select precursors for fragmentation. Endogenous and 2 possible exogenous 1, N^2^-propano-dG structures were respectively targeted at *m/z* of 338.1450, 340.1526 (+2 form exogenous), and 342.1593 (+4 form exogenous) with co-monitoring of the internal standard ([^13^$C_{10}^{15}$N_5_]- 1, N^2^-propano-dG at *m/z* 353.1651) for fragmentations. The inclusion list for the detection of N^2^-ε-dG was comprised of *m/z* 292.1040 (endogenous N^2^-ε-dG) and *m/z* 302.1375 (internal standard [^13^$C_{5}^{15}$N_5_]-N^2^-ε-dG). The automatic gain control target and the maximum fill time were set at 3 × 10^6^ and 250 ms, respectively. The resolution of the Orbitrap was set at 60 000. The isolation width was set to 1.4 *m*/*z* for the selection of precursors, and the following higher-energy collisional dissociation (HCD) was set to a normalized collision energy of 25 for all targets. The quantification analysis was done by extracting the major product ion with a neutral loss of deoxyribose moiety from each corresponding precursor (Supplementary Table 2). Quantitation of the analytes was achieved by fitting their respective signal ratio against their corresponding internal standard to a prepared calibration curve to obtain the amount ratio, which was then multiplied by the known amount of spiked internal standards and normalized by the amount of deoxyguanosine (dG) measured from the HPLC-UV system. The calibration curve converting signal ratio to amount ratio in the LC-MS/MS analysis was based on our previous studies (Hsiao *et al.*, 2020; Liu *et al.*, 2021).

##### Statistical analysis

Kruskal-Wallis nonparametric test was applied to determine the statistical significance of DNA adduct amounts between the different exposure groups. Dunn’s test with the Benjamini-Hochberg *p*-value adjustment procedure was done to identify group difference, and an adjusted *p*-value was reported. The significant level of tests was set at 0.05. Statistical tests and figures were made on R (ver. 4.0.3) combined with RStudio (ver. 1.4.1103, Boston, MA) or Prism 8 (GraphPad).

## RESULTS

###  

#### Animal Exposure

**Figure 2.**
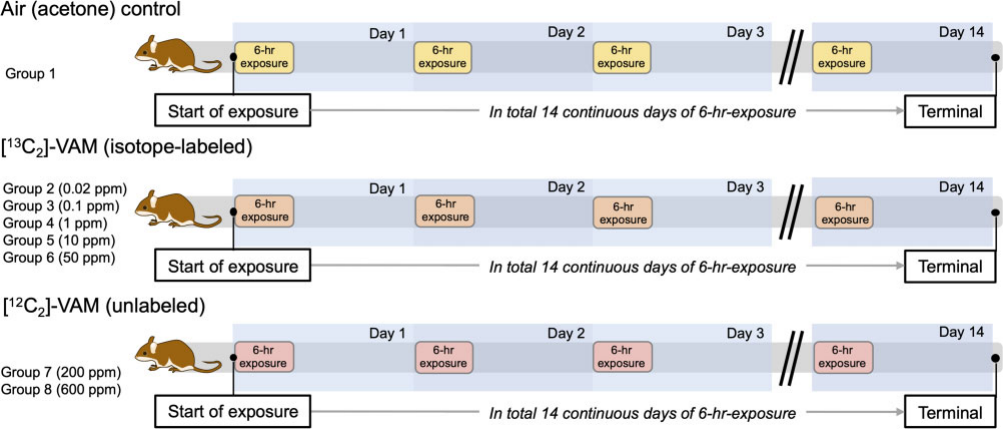
Rats were exposed to VAM for 14 days (6 h/day) using a nose-only inhalation unit. The group 1 was the control, with rats exposed to air (acetone as the vehicle chemical to vaporize VAM through the study). Rats in the groups 2, 3, 4, 5, and 6 were exposed to 0.02, 0.1, 1, 10, and 50 ppm [^13^C_2_]-VAM for 14 days. Rats in the group of 7 and 8 were exposed to 200 and 600 unlabeled [^12^C_2_]-VAM for 14 days.

The animal exposure setup is illustrated in Figure  2. Briefly, rats were exposed to 0.02, 0.1, 1, 10, and 50 ppm stable isotope labeled [^13^C_2_]-VAM; or 200 and 600 ppm unlabeled [^12^C_2_]-VAM for 14 consecutive days (6 h/day). Unlabeled VAM was used for the 200 and 600 ppm exposure groups due to the high cost of using [^13^C_2_]-VAM for 14 days at high concentrations. Acetone was the vehicle chemical used to vaporize VAM in this study, and rats exposed to air/acetone served as the control group and was annotated as group 1. Other groups exposed to VAM were annotated as the exposure groups 2–8 with the dose ranging from 0.02 to 600 ppm. It should be noted that analytically measured concentrations of VAM were generally higher than target ones and used in dose-response analysis in the study, as shown in Table  1.

**Table 1.** Endogenous and Exogenous N^2^-Et-dG Adduct Amount (adducts/10^8^ dG) in Nasal Tissues Including Respiratory and Olfactory Epithelia of Rats Exposed to Various Concentrations of VAM for 6 h/day and 14 Consecutive Days

Tissue	Exposure Group	Exposure Type	Target Concentration (ppm)	Analytically Measured Concentration (ppm)	*n*	[^12^C_2_]-N^2^-Et-dG Level (adduct/10^8^ dG)	[^13^C_2_]-N^2^-Et-dG Level (adduct/10^8^ dG)	Calculated Exogenous N^2^-Et-dG Level (adduct/10^8^ dG)	Exogenous-Endogenous Ratio
Respiratory	1	—	0	0	7	26.03 ± 16.44	N.A.^b^	<0.022	—
	2	[^13^C_2_]-VAM	0.02	0.04	7	22.49 ± 5.69	N.D.^c^	<0.022	<0.00097
					3^a^	31.87 ± 3.31	N.D.^c^	<0.0018	<0.000056
	3	[^13^C_2_]-VAM	0.1	0.23	7	23.82 ± 4.10	N.D.^c^	<0.022	<0.00092
					3^a^	38.94 ± 2.34	N.D.^c^	<0.0018	<0.000046
	4	[^13^C_2_]-VAM	1	2.5	7	23.01 ± 4.82	N.D.^c^	<0.022	<0.00095
					3^a^	19.18 ± 4.15	N.D.^c^	<0.0018	<0.000093
	5	[^13^C_2_]-VAM	10	21	7	26.60 ± 8.25	2.16 ± 0.85	2.16 ± 0.85	0.09 ± 0.05
	6	[^13^C_2_]-VAM	50	54	7	23.43 ± 4.93	15.00 ± 6.02	15.00 ± 6.02	0.67 ± 0.31
	7	Unlabeled VAM	200	210	7	266.36 ± 56.17	N.A.^b^	240.32 ± 56.17	9.23 ± 2.15
	8	Unlabeled VAM	600	626	7	603.83 ± 190.49	N.A.^b^	577.79 ± 190.49	22.19 ± 7.31
Olfactory	1	—	0	0	7	38.21 ± 14.63	N.A.^b^	<0.022	—
	2	[^13^C_2_]-VAM	0.02	0.04	7	40.54 ± 14.51	N.D.^c^	<0.022	<0.00054
					3^a^	43.76 ± 5.74	N.D.^c^	<0.0018	<0.000041
	3	[^13^C_2_]-VAM	0.1	0.023	7	39.14 ± 17.27	N.D.^c^	<0.022	<0.00056
					3^a^	43.70 ± 2.21	N.D.^c^	<0.0018	<0.000041
	4	[^13^C_2_]-VAM	1	2.5	7	38.64 ± 19.10	N.D.^c^	<0.022	<0.00057
					3^a^	31.20 ± 1.87	N.D.^c^	<0.0018	<0.000058
	5	[^13^C_2_]-VAM	10	21	7	38.88 ± 18.87	N.D.^c^	<0.022	<0.00057
					5^a^	20.97 ± 1.96	0.08 ± 0.001	0.08 ± 0.001	0.003 ± 0.007
	6	Unlabeled VAM	50	54	7	36.08 ± 18.83	1.27 ± 0.33	1.27 ± 0.33	0.046 ± 0.027
	7	Unlabeled VAM	200	210	7	81.78 ± 23.20	N.A.^b^	43.57 ± 23.19	1.140 ± 0.607
	8	—	600	626	7	371.09 ± 101.26	N.A.^b^	332.88 ± 101.26	8.712 ± 2.650

Data are presented as mean ± standard deviation.

a DNA from every 3–4 rats were pooled for analysis (*n *=* *3 or 5) to improve sensitivity for low dosing groups of 0.02, 0.1,1, and 10 ppm in addition to individual rat samples analyzed (*n *=* *7). In total, DNA from approximately 20 rats were used in each dosing group for adduct analysis.

b N.A.: not exposed to [^13^C_2_]-VAM. ^13^C adducts were not expected and detected in these samples.

c N.D.: exposed to [^13^C_2_]-VAM but ^13^C adducts not detected (ie, below the LOD).

#### Analytical Assay for DNA Adduct Quantification

The primary goal of this study was to establish the molecular dosimetry of N^2^-EtD-dG DNA adducts in rats exposed to VAM. Various doses of stable isotope labeled [^13^C_2_]-VAM were exposed to rats through nose-only inhalation units to distinguish exogenous [^13^C_2_]-N^2^-Et-dG adducts from endogenous adducts. Exogenous [^13^C_2_]-N^2^-EtD-dG and endogenous N^2^-EtD-dG in DNA were chemically reduced to [^13^C_2_]-N^2^-Et-dG and N^2^-Et-dG, respectively (Matsuda *et al.*, 2007; Mizumoto *et al.*, 2017; Moeller *et al.*, 2013). The mass spectrometry-based analytical platform for accurate quantification of N^2^-Et-dG, N^2^-ε-dG, and 1, N^2^-propano-dG adducts has been demonstrated in our previous studies (Hsiao *et al.*, 2020; Liu *et al.*, 2021).

**Figure 3.**
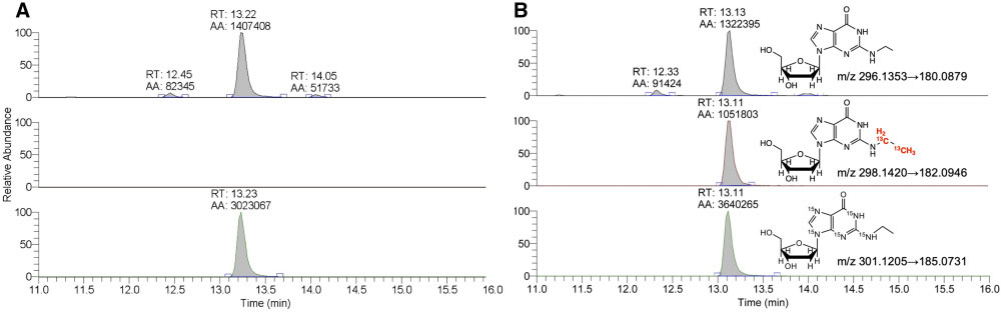
Representative nano-LC-ESI-MS/MS PRM chromatograms of endogenous N^2^-Et-dG (upper panel), exogenous [^13^C_2_]-N^2^-Et-dG (middle panel), and [^15^N_5_]-N^2^-Et-dG spiked in samples as internal standard (bottom panel) in nasal respiratory epithelium of rats exposed to acetone (as control vehicle) (A) and 50 ppm [^13^C_2_]-VAM (B). Chemical structures of N^2^-Et-dG, [^13^C_2_]-N^2^-Et-dG, and [^15^N_5_]-N^2^-Et-dG and their quantifying transition were annotated in each panel. Quantifying product ion of each precursor was the major fragment ion with a neutral loss of deoxyribose moiety after fragmentation.

Figure  3 shows representative nano-LC-ESI-MS/MS PRM chromatograms of endogenous N^2^-Et-dG, exogenous [^13^C_2_]-N^2^-Et-dG, and [^15^N_5_]-N^2^-Et-dG internal standard in nasal respiratory epithelium of rats exposed to air control and 50 ppm [^13^C_2_]-VAM. Clearly, in the control rats, exogenous [^13^C_2_]-N^2^-Et-dG adduct was not detected. Signal ratios between the analyte (endogenous or exogenous adduct signals) and internal standard were used to calculate the exact amounts of target analytes based on the known amount of spiked internal standard. The final amounts of target analytes were normalized to the dG amounts of samples used for HPLC purification after DNA digestion. The quantification of N^2^-ε-dG and 1, N^2^-propano-dG followed the same strategy in calculation. The on-column limit of detection for N^2^-Et-dG, N^2^-ε-dG, and 1, N^2^-propano-dG on the LC-MS/MS platform were 1.2, 4.7, and 6.0 amol, respectively. Our previous study demonstrated that N^2^-EtD-dG was the primary exogenous DNA adducts induced by VAM exposure, with exogenous 1, N^2^-propano-dG only being detected in rats exposed to a single exposure of 400 ppm [^13^C_2_]-VAM for 6 h (Liu *et al.*, 2021). Herein, exogenous 1, N^2^-propano-dG adducts were not detected in nasal epithelium of rats exposed to 50 ppm [^13^C_2_]-VAM for 14 days (Supplementary Figure 2), whereas exogenous N^2^-EtD-dG adducts were readily detected in the same exposed rats, again highlighting N^2^-EtD-dG as the primary exogenous DNA adducts of VAM. Consequently, the focus of our analysis and discussion has been placed on N^2^-EtD-dG adducts.

#### Molecular Dosimetry of Exogenous N^2^-Et-dG Adducts

The results for endogenous and exogenous N^2^-EtD-dG (detected as N^2^-Et-dG) measurement in the rat nasal respiratory and olfactory epithelia are summarized in Table  1. Endogenous N^2^-Et-dG adducts were detected in all nasal epithelium tissues we analyzed, with the amount of endogenous adduct of approximately 30 adducts/10^8^ dG. Exogenous [^13^C_2_]-N^2^-Et-dG were detected in rats exposed to 10 and 50 ppm [^13^C_2_]-VAM. As unlabeled [^12^C_2_]-VAM was used for 200 and 600 ppm groups, no ^13^C-adducts derived from VAM were expected in these rats. However, the formation of exogenous adducts could be determined by subtracting the background levels of endogenous DNA adducts from the total adducts. As listed in Table  1, normalized exogenous DNA adducts were 240.32 ± 56.17, and 577.79 ± 190.49 adducts/10^8^ dG in the respiratory epithelium of rats exposed to 200 and 600 ppm [^12^C_2_]-VAM, respectively. No exogenous DNA adduct was detected in rats exposed to [^13^C_2_]-VAM at 1 ppm and below, although endogenous N^2^-Et-dG adducts were ubiquitously present in all samples we analyzed.

It should be noted that our capability to detect exogenous DNA adducts also depends on the amount of DNA used for digestion and analysis. Higher amounts of DNA used would increase the likelihood to detect low abundant adducts after adduct enrichment with HPLC. In addition to the data collected using 10 µg DNA, we also pooled samples to get 120 µg DNA for adduct measurement for the dosing groups of 0.02, 0.1, and 1 ppm. However, exogenous [^13^C_2_]-N^2^-Et-dG were still not detected despite 12 times more DNA being used. Under such scenario, the limits of detection were used to estimate the normalized exogenous DNA adducts, as listed in Table  1.

**Figure 4.**
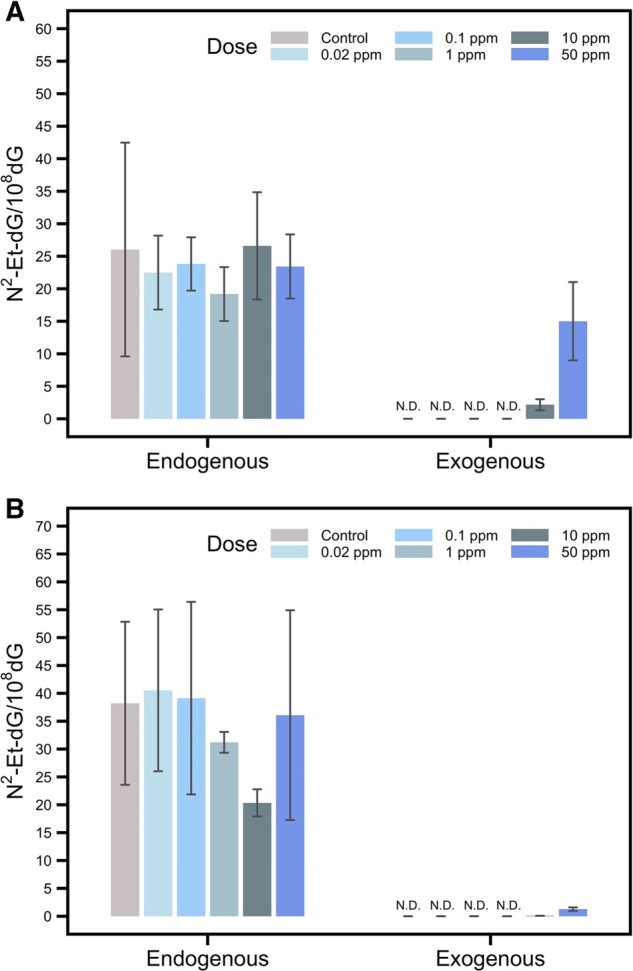
Endogenous (^12^C) and exogenous (^13^C) N^2^-Et-dG in nasal tissues including respiratory epithelium (A) and olfactory epithelium (B). Data were shown in means and standard deviations. Endogenous N^2^-Et-dG and exogenous [^13^C_2_]-N^2^-Et-dG from nasal tissues are analyzed by LC-MS/MS. Olfactory epithelium under exposure to 1 ppm and 10 ppm [^13^C_2_]-VAM were analyzed for 120 µg DNA with *n *=* *3 or 5 replicates. Other tissues are analyzed for 10 µg with *n *=* *7 replicates. Nondetected groups were annotated with “N.D.”

Figure  4 illustrates the levels of endogenous and exogenous N^2^-Et-dG in nasal respiratory and olfactory epithelia of rats exposed to 0.02 to 50 ppm [^13^C_2_]-VAM. Endogenous N^2^-Et-dG was detected in all samples analyzed and showed no difference among groups (*p *=* *.77). This indicates that exposure to [^13^C_2_]-VAM even up to 50 ppm for 14 days did not alter endogenous N^2^-Et-dG. Exogenous [^13^C_2_]-N^2^-Et-dG was only detected in rats exposed to 10 and 50 ppm [^13^C_2_]-VAM. In addition, the amounts of exogenous N^2^-Et-dG were less than endogenous DNA adducts, especially at low concentrations. For example, the ratio of exogenous/endogenous DNA adducts was approximately 0.046 and 0.67 in nasal olfactory and respiratory epithelia of rats exposed to 50 ppm [^13^C_2_]-VAM. Likewise, the ratio of exogenous/endogenous DNA adducts was only approximately 0.0038 and 0.09 in nasal olfactory and respiratory epithelia of rats exposed to 10 ppm [^13^C_2_]-VAM. These data indicate that endogenous DNA adducts were predominant in rats exposed to up to 50 ppm VAM. The exogenous adducts induced by VAM only accounted for a very small portion of total DNA adducts in exposed rats at low doses.

**Figure 5.**
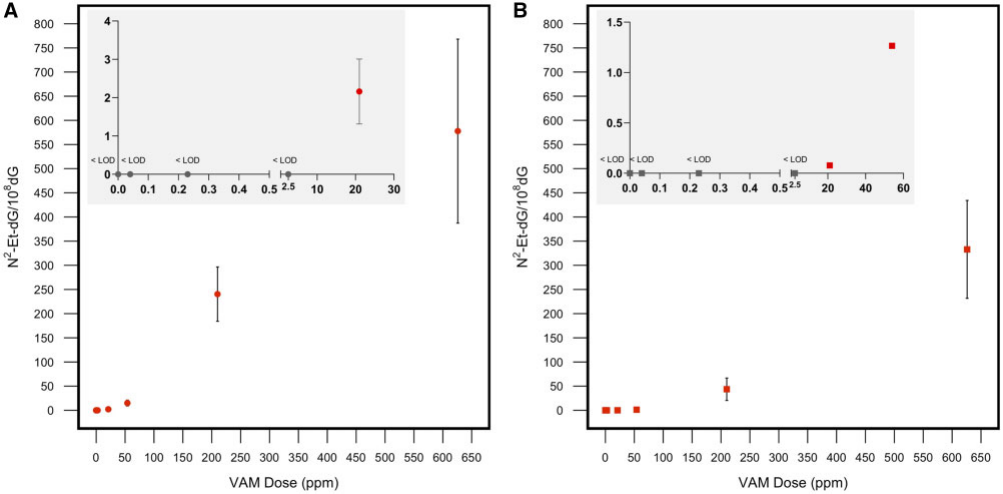
Exogenous N^2^-Et-dG adducts form in a nonlinear fashion in nasal tissues including respiratory epithelium (A) and olfactory epithelium (B). Data were shown in means and standard deviations. Nondetected groups were annotated with “<LOD” (lower than limit of detection of 0.0018 adduct/10^8^ dG).

The dose-response between inhaled concentrations of VAM and the amounts of resulted exogenous N^2^-Et-dG in nasal tissues is presented in Figure  5. A clear nonlinear dose response was observed across the doses used in this study. Consistent with our previous 1-day exposure study (Liu *et al.*, 2021), more exogenous N^2^-Et-dG adducts were observed in respiratory than in olfactory epithelia. However, differential dose-response was observed between nasal respiratory and olfactory epithelia. For instance, the amount of exogenous DNA adducts increased approximately 38.5- and 262-fold in nasal respiratory and olfactory epithelia respectively, when the dose was increased from 50 to 600 ppm.

#### Potential Systemic Distribution of VAM or Its Metabolite

**Figure 6.**
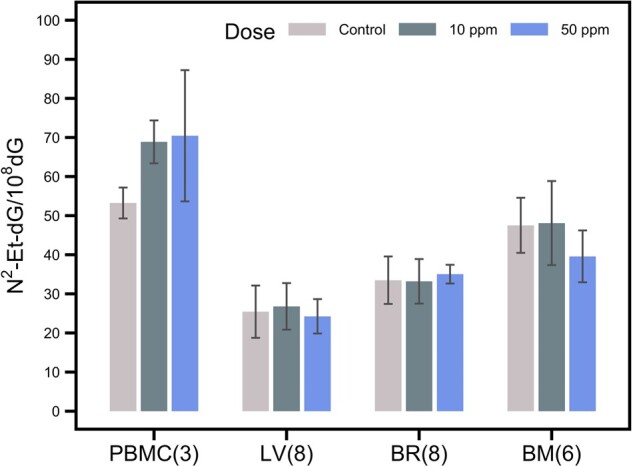
Measurement of endogenous N^2^-Et-dG in peripheral blood mononuclear cells (PBMC), liver (LV), brain (BR), and bone marrow (BM) of rats exposed to air control, 10 ppm [^13^C_2_]-VAM, and 50 ppm [^13^C_2_]-VAM. Data were shown in means and standard deviations. Sample size (*n*) were annotated in the beside the types of tissue. Levels of endogenous N^2^-Et-dG levels in each tissue show no statistical significance of difference by Kruskal-Wallis tests.

In addition to DNA adducts at respiratory and olfactory epithelia, VAM or its metabolite may potentially further distribute and result in adducts in distant organs. Other tissues including PBMC, liver, brain, and bone marrow were also analyzed for endogenous and exogenous N^2^-Et-dG to examine potential systemic distribution of VAM. We particularly focused on the dosing groups in which exogenous DNA adducts were detected in the nasal epithelium. PBMC from group 1 (control) and groups 5–8 (10 and 50 ppm [^13^C_2_]-VAM, 200 and 600 ppm [^12^C_2_]-VAM) were examined. Livers, brains, and bone marrows of rats in group 1 (control), group 5 (10 ppm), and group 6 (50 ppm) were also analyzed. Table  2 summarized the results of endogenous and exogenous N^2^-Et-dG in PBMC samples of rats. Exogenous [^13^C_2_]-N^2^-Et-dG was undetectable in the control group (group 1) and in rats exposed to 10 ppm [^13^C_2_]-VAM (group 5). Exogenous N^2^-Et-dG was detected in 1 of 3 pooled replicates of PBMC samples in the group 6 rats (50 ppm [^13^C_2_]-VAM), with approximately 0.21 adducts/10^8^ dG. The level of exogenous adduct was only 0.19% of the endogenous adduct measured in the same sample. For group 7 (200 ppm [^12^C_2_]-VAM) and group 8 (600 ppm [^12^C_2_]-VAM), the measured N^2^-Et-dG had levels of 37.17 ± 2.38 and 50.63 ± 3.50 adducts/10^8^ dG, which had no statistically significant different (*p *=* *.10 and 0.82, respectively) when compared with the controls. Likewise, no exogenous adduct was detected in liver, brain, and bone marrow samples of rats exposed to [^13^C_2_]-VAM, and no statistically significant difference in endogenous adducts among exposure groups (Figure 6).

**Table 2.** Endogenous and Exogenous N^2^-Et-dG Adduct Amount (adducts/10^8^ dG) in PBMCs of Rats Exposed to Various Concentrations of VAM for 6 h/day and 14 Consecutive Days

Exposure Group	Exposure Type	Target Concentration (ppm)	Analytically Measured Concentration (ppm)	*n* ^a^	[^12^C_2_]-N^2^-Et-dG Level (adduct/10^8^ dG)	[^13^C_2_]-N^2^-Et-dG Level (adduct/10^8^ dG)	Calculated Exogenous N^2^-Et-dG Level (adduct/10^8^ dG)	Exogenous-Endogenous Ratio
1	—	0	0	3	53.23 ± 3.98	N.A.^b^	<0.0018	—
5	[^13^C_2_]-VAM	10	21	3	68.88 ± 5.51	N.D.^c^	<0.0018	<0.0000382
6	[^13^C_2_]-VAM	50	54	3	70.44 ± 16.78	0.2102	0.2102	0.0019
7	Unlabeled VAM	200	210	3	37.17 ± 2.38	N.A.^b^	N.D.	N.A.
8	Unlabeled VAM	600	626	3	50.63 ± 3.50	N.A.^b^	N.D.	N.A.

Data are presented in mean ± standard deviation.

a Sample size (*n*): For PBMC samples, 120 µg DNA was used, and 3 replicates were pooled from every 2–3 rats.

b N.A.: not exposed to [^13^C_2_]-VAM. ^13^C adducts were not expected and detected in these samples.

c N.D.: exposed to [^13^C_2_]-VAM but ^13^C adducts not detected (ie, below the LOD).

#### Detection of N^2^-ε-dG Adducts Among Tissues

The metabolite of VAM, acetaldehyde, may induce ROS that leads to lipid peroxidation, which synthesizes α,β-unsaturated aldehydes to attacks DNA and form the mutagenic N^2^-ε-dG (Mizumoto *et al.*, 2017). Therefore, we further measured the levels of N^2^-ε-dG in the tissue samples of rats exposed to VAM. A representative LC-MS/MS chromatogram of N^2^-ε-dG was shown in Supplementary Figure 3. N^2^-ε-dG was successfully detected in all tissue samples in different exposure groups (Table  3). Levels of N^2^-ε-dG in the nasal tissues including respiratory and olfactory epithelia were similar among all groups, with *p* values of Kruskal-Wallis groupwise test in respiratory and olfactory epithelia being 0.57 and 0.99, respectively. PBMC was analyzed for N^2^-ε-dG in rats of the groups 1, 5, 6, 7, and 8 and showed no statistically significant difference (*p* value = .11). For liver, brain, and bone marrow, N^2^-ε-dG in groups 1, 5, 6 were analyzed and showed no statistically significant difference among exposure groups as well (*p* values were .46, .44, and .45, respectively). Taken together, N^2^-ε-dG was monitored as a biomarker of lipid peroxidation in this study, but no difference was observed among the exposure groups of all tissues analyzed.

**Table 3.** N^2^-ε-dG Adduct Amount (adducts/10^8^ dG) in Tissues of Rats Exposed to Various Concentrations of VAM for 6 h/day and 14 Consecutive Days

Exposure Group	Exposure Type	Target Concentration (ppm)	Analytically Measured Concentration (ppm)^a^	Respiratory Epithelium	Olfactory Epithelium	Peripheral Blood Mononuclear Cell^b^	Liver	Brain	Bone Marrow
1	—	0	0	1.66 ± 0.38 (7)^a^	1.85 ± 0.77 (7)	0.90 ± 0.49 (3)	1.31 ± 0.27 (8)	1.34 ± 0.07 (8)	2.68 ± 0.40 (6)
2	[^13^C_2_]-VAM	0.02	0.04	1.79 ± 0.58 (7)	1.87 ± 1.24 (7)	—	—	—	—
3	[^13^C_2_]-VAM	0.1	0.23	1.65 ± 0.41 (7)	1.78 ± 0.98 (7)	—	—	—	—
4	[^13^C_2_]-VAM	1	2.5	1.84 ± 0.48 (7)	1.87 ± 1.01 (7)	—	—	—	—
5	[^13^C_2_]-VAM	10	21	1.42 ± 0.30 (7)	1.90 ± 1.10 (7)	1.19 ± 0.24 (3)	1.29 ± 0.30 (8)	1.33 ± 0.04 (8)	3.67 ± 1.79 (6)
6	[^13^C_2_]-VAM	50	54	1.43 ± 0.42 (7)	2.03 ± 1.47 (7)	1.45 ± 0.08 (3)	1.21 ± 0.28 (8)	1.29 ± 0.01 (8)	5.04 ± 1.59 (6)
7	Unlabeled VAM	200	210	1.93 ± 0.97 (7)	1.97 ± 1.06 (7)	0.94 ± 0.04 (3)	—	—	—
8	Unlabeled VAM	600	626	1.87 ± 0.71 (7)	2.38 ± 1.98 (7)	1.30 ± 0.08 (3)	—	—	—

a Data are presented as “mean ± standard deviation (number of biological replicates).” A total of 10 µg DNA from each of the tissue was used for analysis if not specified. Tissues not analyzed are annotated with “—.”

b 120 µg DNA pooled from 2 to 3 rats in the same exposure group was used for analysis.

## DISCUSSION

In this study, stable isotope labeled [^13^C_2_]-VAM was utilized for inhalation exposure in rats to distinguish exogenous and endogenous N^2^-EtD-dG adducts. Rat tissues exposed to various doses of VAM for 14 consecutive days (6 h/day) were analyzed to quantify exogenous and endogenous DNA adducts. Our objective in the study is to establish the molecular dosimetry of DNA adducts in rats exposed to VAM, which will provide critical new data to improve science-based cancer risk assessment of VAM exposure.

VAM is metabolized into acetaldehyde that can form different DNA adducts including N^2^-EtD-dG and 1, N^2^-propano-dG. In addition, acetaldehyde can cause lipid peroxidation, leading to the formation of etheno-adducts, such as N^2^-ε-dG. Therefore, all 3 DNA adducts were monitored in this study. As described in the Results, exogenous N^2^-EtD-dG adducts were detected in rats exposed to VAM at the concentrations of 10 ppm and above and a nonlinear dose response was observed. However, no exogenous 1, N^2^-propano-dG were detected in the same animal samples. 1, N^2^-propano-dG was only detected in rats exposed to 400 ppm [^13^C_2_]-VAM (6 h/day, 1 day) in our previous study (Liu *et al.*, 2021), however was not detected in rats exposed to 50 ppm [^13^C_2_]-VAM (6 h/day for 14 days) in this study, the highest dosing group using stable isotope labeled VAM for exposure. These results are consistent with our previous study and support that N^2^-EtD-dG is the primary DNA adducts induced by VAM exposure (Liu *et al.*, 2021). Likewise, N^2^-ε-dG adducts were detected in all rat tissues we analyzed, but no statistical significance was found between the control and exposed rats. Taken together, N^2^-EtD-dG may serve as a more sensitive and suitable biomarker to evaluate the exposure and/or effects of VAM exposure. In addition, the nonlinear dose response of N^2^-EtD-dG observed in this study would be useful to model the formation of DNA adducts induced by VAM and in the risk assessment of VAM carcinogenicity.

In this study, rats were exposed to VAM at 0.02, 0.1, 1, 10, 50, 200, and 600 ppm for 14 days (6 h/day), followed by DNA adduct analysis. Exogenous N^2^-Et-dG adducts were found in nasal tissues in rats at 10, 50, 200, and 600 ppm dosing groups (Table  1). Exogenous N^2^-Et-dG was not detectable in nasal epithelium tissues of rats exposed to the doses of 0.02, 0.1, and 1 ppm. Despite 12-fold more DNA used for analysis to increase the chance of detecting exogenous DNA adducts in these low dosing group, no exogenous DNA adducts could be detected. Therefore, for the dosing groups of 0.02, 0.1, and 1 ppm, the limit of detection was used to calculate normalized exogenous DNA adducts and the ratio of exogenous/endogenous DNA adducts. If the exogenous DNA adducts were present in these samples, the normalized number of exogenous DNA adducts should be less than 0.00018 adducts/10^8^ dG in rats exposed to VAM less than 1 ppm. Meanwhile, the amount of endogenous N^2^-Et-dG adducts is approximately 30 adducts/10^8^ dG in the rats. Obviously, the endogenous N^2^-Et-dG DNA adducts predominated at low dosing groups, with the exogenous/endogenous DNA adducts ratios being less than 0.000046 to 0.000093 in rats exposed to 0.02, 0.1, and 1 ppm VAM for 14 days. Our data show that exogenous adducts were less than endogenous DNA adducts even with the dose up to 50 ppm. For example, the ratio of exogenous/endogenous adducts in the respiratory epithelium of rats exposed to 50 ppm was 0.09 ± 0.05 and 0.67 ± 0.31, respectively. Interestingly, exogenous DNA adducts were significantly increased at high doses such as 200 and 600 ppm, with the ratios of exogenous/endogenous adducts being 9.23 ± 2.15 and 22.19 ± 7.31.

This study has demonstrated a clear nonlinear dose response for exogenous N^2^-Et-dG adducts over the dose range of 0.02, 0.1, 1, 10, 50, 200, and 600 ppm. Using the exogenous adducts in respiratory epithelium as an example, the amount of exogenous N^2^-Et-dG adducts increased approximately 7-fold when the dose increased from 10 to 50 ppm group (2.16 ± 0.85 and 15.00 ± 6.02 adducts/10^8^ dG for 10 and 50 ppm, respectively, Table  1). However, the exogenous DNA adducts increased more than approximately 100-fold (240.32 ± 56.17 adducts/10^8^ dG for 200 ppm) while the concentration increased from 10 to 200 ppm. Similar nonlinear dose response was also observed for DNA adducts in olfactory epithelium. More research is needed to delineate the factors that lead to the nonlinear does response. Saturation of DNA repair capacity at high doses may play a role. These data indicate that nonlinear dose response model should be employed when extrapolating results from high doses of exposure to human relevant concentrations. Moreover, molecular dosimetry data we generated in this study may lay the foundation to develop suitable dose-response models to improve science-based risk assessment of VAM.

Our data shows that [^13^C_2_]-VAM induced DNA adducts in the nasal cavity but not distant tissues, which is consistent with the most significant histopathological changes observed in nasal tissues of rats and mice exposed to 50, 200, and 600 ppm VAM for 102 weeks in the previous study; moreover, the majority of tumors were found in olfactory epithelium of rodents at 600 ppm (Bogdanffy *et al.*, 1994). Therefore, the formation of exogenous DNA adducts in both nasal respiratory and olfactory epithelium of exposed rats support the biological plausibility that VAM causes cancer at high concentrations. Consistent with our earlier 1-day kinetics study, we noticed that the exogenous DNA adducts were higher in nasal respiratory epithelium than olfactory epithelium, which may be the consequences of multiple factors, such as diffusion and vapor deposition efficiency of VAM in the nasal cavity, metabolism of VAM, heterogeneity between nasal tissues, and DNA repair, as we already discussed elsewhere (Liu *et al.*, 2021). It has been documented that the deposition efficiency of VAM in the rat upper respiratory tract during inhalation exposure is concentration dependent and is highly nonlinear. Also, CE and the ALDH family are shown to be more active in respiratory than in olfactory epithelia of rats (Bogdanffy *et al.*, 1998). Interestingly, the concentrations of VAM seems play an important role in determining the DNA adduct levels in nasal respiratory and olfactory epithelium. For example, the amount of exogenous N^2^-Et-dG in nasal respiratory epithelium of rats exposed to 50 ppm VAM is approximately 12-fold higher than that of olfactory epithelium (15.00 ± 6.02 and 1.27 ± 0.33 adducts/10^8^ dG, respectively). However, in rats exposed to 600 ppm VAM, the amount of exogenous N^2^-Et-dG in nasal respiratory epithelium is only approximately 1.7-fold higher than olfactory epithelium (577.79 ± 190.49 and 332.88 ± 101.26 adducts/10^8^ dG, respectively). The significant reduced difference in the amounts of exogenous DNA adducts between nasal olfactory and respiratory epithelium at high doses may be impacted by diffusion and vapor deposition efficiency along nasal cavity and potential saturation of metabolism enzymes such as CE and ALDH in nasal respiratory epithelium at high VAM concentrations.

It has been reported that chronic inhalation exposure induced nasal tumor in rodents at high doses (such as 600 ppm), with nasal olfactory epithelium being more sensitive than respiratory epithelium (Albertini, 2013; Bogdanffy *et al.*, 1994, 1997; Budinsky *et al.*, 2013; Morris, 1997; Stanek and Morris, 1999). Herein, we noticed that a significant amount of exogenous N^2^-Et-dG was formed in nasal olfactory epithelia of rats exposed to 600 ppm VAM, which may contribute to the increased tumor incidence in nasal olfactory epithelium. The olfactory epithelium may be more susceptible to tumorigenesis due to its higher rate in cell proliferation. Bogdanffy *et al.* observed the cellular adaptation by evaluating the cell proliferation in the rat nasal respiratory and olfactory epithelia exposed to 0, 50, 200, 600, or 1000 ppm VAM for 1, 5, or 20 days (6 h/day) (Bogdanffy *et al.*, 1997). The trends of cell proliferation were similar between respiratory and olfactory epithelia after 1 and 5 day exposure, however, much more cell proliferation was observed in the olfactory epithelium after 20 days exposure, resulting in the cellular damages mainly confined in the olfactory epithelium. The formation of high amount of exogenous DNA adducts (approximately 8.7-fold higher than endogenous ones), coupled with enhanced cell proliferation, may account for the sensitivity of nasal olfactory in VAM-induced nasal cancer.

There are several limitations associated with the study. First, we were not able to use [^13^C_2_]-VAM for all dosing groups, as [^13^C_2_]-VAM is cost prohibitive for a 14-day high dose exposure. In the dosing groups of 50 ppm and below, [^13^C_2_]-VAM was used, and endogenous and exogenous DNA adducts were unambiguously differentiated and determined by mass spectrometry. The exogenous adducts in the groups of 200 and 600 ppm were calculated by subtracting the amount of endogenous DNA adducts of the controls from the total adducts of rats exposed to 200 and 600 ppm unlabeled VAM. Although such a strategy may not be optimal, it may still offer a reasonable method to estimate exogenous DNA adducts induced by exposure, as our data indicate that VAM exposure does not alter the amount of endogenous DNA adducts. Second, systemic distribution of VAM can exist, especially under high concentrations of exposure. We observed insignificant difference for N^2^-Et-dG in the PBMC samples in the 200 and 600 ppm groups compared with nonexposed controls. However, the lack of isotopic labels hindered us from differentiating endogenous and exogenous N^2^-Et-dG, especially when the exogenous adducts existed in a trace amount thus were veiled by the abundant endogenous adducts. Third, we exposed rats to VAM for 14 days (6 h/day). The exposure period may not be enough to approach the steady state of exogenous DNA adducts yet and a longer exposure time, such as 28 days, would be more appropriate. However, we have generated data on DNA adducts from 1 day exposure and 14-day exposure, coupled with the kinetics of DNA adduct repair/half-life from our previous study (Liu *et al.*, 2021), which would allow the estimation of the steady state of exogenous DNA adducts to improve science-based cancer risk assessment of VAM.

## SUPPLEMENTARY DATA

Supplementary data are available at *Toxicological Sciences* online.

## FUNDING

This project received financial sponsorship from the Vinyl Acetate Council. The sponsor has not been involved in writing or has no access to the manuscript before its publication.

## DECLARATION OF CONFLICTING INTERESTS

The authors declared no potential conflicts of interest with respect to the research, authorship, and/or publication of this article.

## Supplementary Material

kfab140_Supplementary_Data

## References

[kfab140-B1] Albertini R. J. (2013). Vinyl acetate monomer (VAM) genotoxicity profile: Relevance for carcinogenicity. Crit. Rev. Toxicol. 43, 671–706. 23985073 10.3109/10408444.2013.827151

[kfab140-B2] Beland F. A. , Churchwell M. I., Von Tungeln L. S., Chen S., Fu P. P., Culp S. J., Schoket B., Gyorffy E., Minárovits J., Poirier M. C., et al (2005). High-performance liquid chromatography electrospray ionization tandem mass spectrometry for the detection and quantitation of benzo[a]pyrene−DNA adducts. Chem. Res. Toxicol. 18, 1306–1315. 16097804 10.1021/tx050068y

[kfab140-B3] Bogdanffy M. S. , Dreef-van der Meulen H. C., Beems R. B., Feron V. J., Cascieri T. C., Tyler T. R., Vinegar M. B., Rickard R. W. (1994). Chronic toxicity and oncogenicity inhalation study with vinyl acetate in the rat and mouse. Fundam. Appl. Toxicol. 23, 215–229. 7982530 10.1006/faat.1994.1100

[kfab140-B4] Bogdanffy M. S. , Gladnick N. L., Kegelman T., Frame S. R. (1997). Four-week inhalation cell proliferation study of the effects of vinyl acetate on rat nasal epithelium. Inhal. Toxicol. 9, 331–350.

[kfab140-B5] Bogdanffy M. S. , Sarangapani R., Kimbell J. S., Frame S. R., Plowchalk D. R. (1998). Analysis of vinyl acetate metabolism in rat and human nasal tissues by an in vitro gas uptake technique. Toxicol. Sci. 46, 235–246. 10048126 10.1006/toxs.1998.2542

[kfab140-B6] Bogdanffy M. S. , Sarangapani R., Plowchalk D. R., Jarabek A., Andersen M. E. (1999). A biologically based risk assessment for vinyl acetate-induced cancer and noncancer inhalation toxicity. Toxicol. Sci. 51, 19–35. 10496674 10.1093/toxsci/51.1.19

[kfab140-B7] Budinsky R. , Gollapudi B., Albertini R. J., Valentine R., Stavanja M., Teeguarden J., Fensterheim R., Rick D., Lardie T., McFadden L., et al (2013). Nonlinear responses for chromosome and gene level effects induced by vinyl acetate monomer and its metabolite, acetaldehyde in tk6 cells. Environ. Mol. Mutagen. 54, 755–768. 24038327 10.1002/em.21809

[kfab140-B8] Chatterjee N. , Walker G. C. (2017). Mechanisms of DNA damage, repair, and mutagenesis. Environ. Mol. Mutagen. 58, 235–263. 28485537 10.1002/em.22087 PMC5474181

[kfab140-B9] Hsiao Y.-C. , Liu C.-W., Chi L., Yang Y., Lu K. (2020). Effects of gut microbiome on carcinogenic DNA damage. Chem. Res. Toxicol. 33, 2130–2138. 32677427 10.1021/acs.chemrestox.0c00142 PMC7839797

[kfab140-B10] IARC. (1987). Overall evaluations of carcinogenicity: An updating of IARC monographs volumes 1 to 42. In International Agency for Research on Cancer WHO, IARC Working Group on the Evaluation of Carcinogenic Risks to Humans, editor. International Agency for Research on Cancer, Lyon. 3482203

[kfab140-B11] Jantunen K. , Mäki-Paakkanen J., Norppa H. (1986). Induction of chromosome aberrations by styrene and vinylacetate in cultured human lymphocytes: Dependence on erythrocytes. Mutat. Res. 159, 109–116. 3941660 10.1016/0027-5107(86)90119-3

[kfab140-B12] Kuykendall J. R. , Taylor M. L., Bogdanffy M. S. (1993). Cytotoxicity and DNA-protein crosslink formation in rat nasal tissues exposed to vinyl acetate are carboxylesterase-mediated. Toxicol. Appl. Pharmacol. 123, 283–292. 8248935 10.1006/taap.1993.1247

[kfab140-B13] Lambert B. , Chen Y., He S. M., Sten M. (1985). DNA cross-links in human leucocytes treated with vinyl acetate and acetaldehyde in vitro. Mutat. Res. 146, 301–303. 4058447 10.1016/0167-8817(85)90072-0

[kfab140-B14] Liu C. W. , Hsiao Y. C., Hoffman G., Lu K. (2021). LC-MS/MS analysis of the formation and loss of DNA adducts in rats exposed to vinyl acetate monomer through inhalation. Chem. Res. Toxicol. 34, 793–803. 33486946 10.1021/acs.chemrestox.0c00404

[kfab140-B15] Matsuda T. , Matsumoto A., Uchida M., Kanaly R. A., Misaki K., Shibutani S., Kawamoto T., Kitagawa K., Nakayama K. I., Tomokuni K., et al (2007). Increased formation of hepatic n2-ethylidene-2'-deoxyguanosine DNA adducts in aldehyde dehydrogenase 2-knockout mice treated with ethanol. Carcinogenesis 28, 2363–2366. 17361010 10.1093/carcin/bgm057

[kfab140-B16] Mizumoto A. , Ohashi S., Hirohashi K., Amanuma Y., Matsuda T., Muto M. (2017). Molecular mechanisms of acetaldehyde-mediated carcinogenesis in squamous epithelium. Int. J. Mol. Sci. 18, 1943. 10.3390/ijms18091943 PMC5618592 28891965

[kfab140-B17] Moeller B. C. , Recio L., Green A., Sun W., Wright F. A., Bodnar W. M., Swenberg J. A. (2013). Biomarkers of exposure and effect in human lymphoblastoid tk6 cells following [13c2]-acetaldehyde exposure. Toxicol. Sci. 133, 1–12. 23425604 10.1093/toxsci/kft029 PMC3627555

[kfab140-B18] Morris J. B. (1997). Uptake of acetaldehyde vapor and aldehyde dehydrogenase levels in the upper respiratory tracts of the mouse, rat, hamster, and guinea pig. Fundam Appl. Toxicol. 35, 91–100. 9024677 10.1006/faat.1996.2263

[kfab140-B19] Owen P. E. (1988). Vinyl acetate: 104 week inhalation combined chronic toxicity and carcinogenicity study in the rat and mouse. Hazleton Laboratories Europe Ltd, Harrogate, England; England for the Society of the Plastics Industry. No. 5547-51/15.

[kfab140-B20] Rundle A. (2006). Carcinogen-DNA adducts as a biomarker for cancer risk. Mutat. Res. Fundam. Mol. Mech. Mutagenesis 600, 23–36. 10.1016/j.mrfmmm.2006.05.031 16824556

[kfab140-B21] Stanek J. J. , Morris J. B. (1999). The effect of inhibition of aldehyde dehydrogenase on nasal uptake of inspired acetaldehyde. Toxicol. Sci. 49, 225–231. 10416267 10.1093/toxsci/49.2.225

