# Supplementary material for: Molecular Dosimetry of DNA Adducts in Rats Exposed to Vinyl Acetate Monomer
Source: Toxicol Sci. 2021 Dec 14;185(2):197–207. doi: 10.1093/toxsci/kfab140 (PMC8795904; doi:10.1093/toxsci/kfab140)
Supplement: kfab140_Supplementary_Data [file kfab140_supplementary_data.docx]

**<< Supporting Information >>**

**Molecular Dosimetry of DNA Adducts in Rats Exposed to Vinyl Acetate Monomer**

**Authors:** Yun-Chung Hsiao^†1^, Chih-Wei Liu^†1^, Gary Hoffman^2^, Kun Lu^1*^

^†^ Contributed equally to this work

**Affiliations:**

^1^ Department of Environmental Sciences and Engineering, University of North Carolina at Chapel Hill, Chapel Hill, North Carolina 27599, United States

^2^ Covance CRS, LLC, Somerset, New Jersey 08873, United States

**Corresponding Author:** Dr. Kun Lu

**Address:** Department of Environmental Sciences and Engineering, University of North Carolina at Chapel Hill, Chapel Hill, North Carolina 27599, United States

**Email:** [kunlu@unc.edu](mailto:kunlu@unc.edu)

**Telephone:** 919-966-7377

**Table of Contents**

**Table S1.** Method summary for gas chromatography-flame ionization detector detection. ……………………**S3**

**Figure S1.** UV-signal to dG amount calibration curve on HPLC-UV system for dG quantification. ………….**S4**

**Figure S2.** Representative nano-LC-ESI-MS/MS PRM chromatograms for the detection of 1,N^2^-propano-dG and its corresponding internal standard in samples of this study. ………………………………………………**S5**

**Figure S3.** Representative nano-LC-ESI-MS/MS PRM chromatograms for the detection of N^2^-ε-dG and its corresponding internal standard in samples of this study. ………………………………………………………**S6**

**Table S2.** Mass accuracy of DNA adducts measured in Q Exactive HF mass spectrometry. …………………..**S7**

**Table S1.** Method summary for gas chromatography-flame ionization detector detection.

| Parameter | Value of parameter |
| --- | --- |
| Test item | Vinyl acetate |
| Matrix | Charcoal tubes |
| Calibrarion range | 2.00 μg/mL to 20 μg/mL |
| Regression type | Linear |
| Data calculation | Peak area |
| Limit of quantification | 2.00 μg/mL |
| Analytical instrumentation | GC (Agilent Technologies) |
| Type of detection | GC-FID (Gas Chromatography – Flame Ionization Detector Detection) |
| Column type | Zebron ZB-5 (Phenomenex) |
| Column dimensions | (30 m length × 0.32 mm inner diameter), 0.25 μm film thickness |
| Run time | 5.5 minutes |
| Carrier gas | H2 |
| Carrier gas mode | Constant flow |
| Carrier gas flow | 2.00 mL/min |
| Needle rinse solution | Methylene chloride |
| Inlet liner | Agilent Inlet liner, split, focus, glass wool, deactivated (Part #: 210-4004-5) |
| Injection volume | 1.5 μL |
| Inlet temperature | 210°C |
| Inlet mode | Split |
| Split ratio | 10 |
| Detector temperature | 300°C |
| Detector range | 0 |
| Detector H2 flow | 40.00 mL/min |
| Detector air flow | 400.00 mL/min |
| Detector N2 flow | 30.00 mL/min |
| Extraction solvent | 100% Methylene chloride |
| Temperature program | \| Initial temperature (°C) \| ΔT (°C/min) \| Final temperature (°C) \| Ramp time (min) \| Hold time (min) \| \| --- \| --- \| --- \| --- \| --- \| \| 32.0 \| NA \| NA \| NA \| 3.00 \| \| 32.0 \| 6.0 \| 35.0 \| 0.5 \| 2.00 \|   aa |


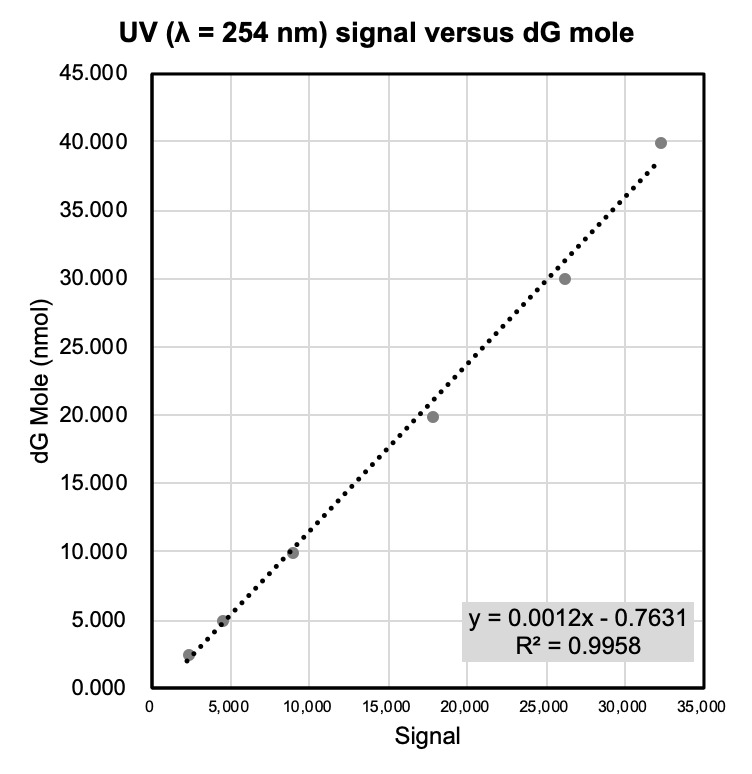


**Figure S1.** UV-signal to dG amount calibration curve on HPLC-UV system for dG quantification. The linear regression equation obtained (UV signal = 0.0012 × dG amount – 0.7631) can be used to measure dG amount in a sample.

**
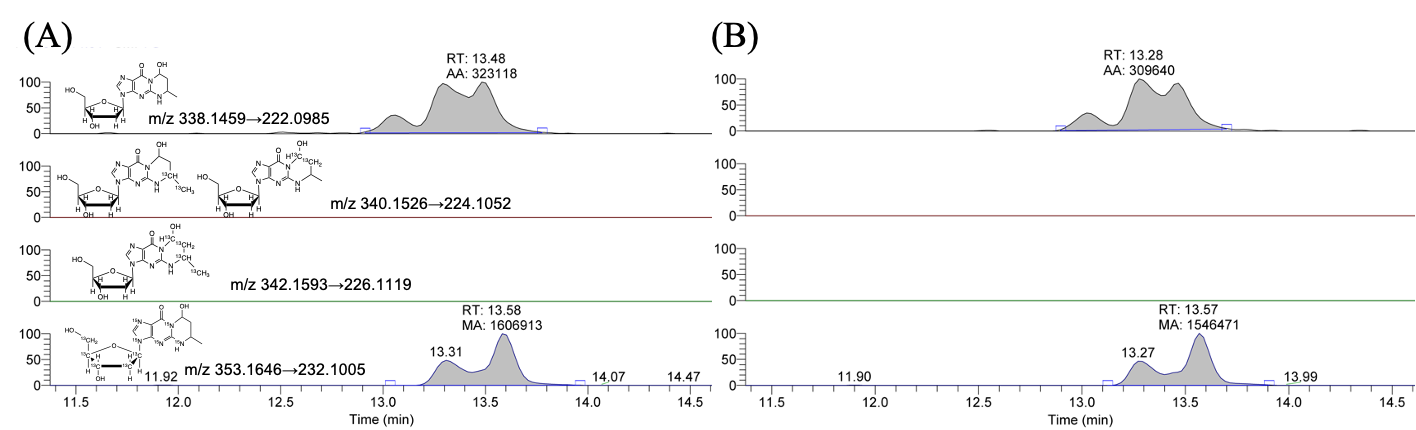
**

**Figure S2.** Representative nano-LC-ESI-MS/MS PRM chromatograms for the detection of 1,N^2^-propano-dG and its corresponding internal standard in samples of this study. The four panels from the upper to the lower refer to native (endogenous) 1,N^2^-propano-dG, exogenous [^13^C_2_]-1,N^2^-propano-dG, exogenous [^13^C_4_]-1,N^2^-propano-dG, and the spiked internal standard [^13^C_10_^15^N_5_]-1,N^2^-propano-dG in respiratory and olfactory epithelia exposed to 50 ppm [^13^C_2_]-VAM for 6 hr/day, 14 continuous days.

**Figure S3.** Representative nano-LC-ESI-MS/MS PRM chromatograms for the detection of N^2^-ε-dG and its corresponding internal standard in samples of this study. The upper panel refers to N^2^-ε-dG detected in sample and the lower panel refers to the spiked internal standard [^13^C_10_^15^N_5_]- N^2^-ε-dG.

**Table S2.** Mass accuracy of DNA adducts measured in Q Exactive HF mass spectrometry.

| Analyte | | | | |  | Internal standard | | | | |
| --- | --- | --- | --- | --- | --- | --- | --- | --- | --- | --- |
| Adducts**^A^** | Ion type | Measured m/z | Theoretical m/z | Mass accuracy (ppm) |  | Adducts**^A^** | Ion type | Measured  m/z | Theoretical  m/z | Mass accuracy (ppm) |
| N^2^-Et-dG | Precursor ion | 296.13555 | 296.13533 | 0.74 |  | [^15^N_5_]-N^2^-Et-dG | Precursor ion | 301.12084 | 301.12051 | 1.10 |
|  | Product ion | 180.08800 | 180.08799 | 0.06 |  |  | Product ion | 185.07325 | 185.07316 | 0.49 |
| [^13^C_2_]-N^2^-Et-dG | Precursor ion | -**^B^** | 298.14204 | - |  |  |  |  |  |  |
|  | Product ion | 182.09496 | 182.09470 | 1.42 |  |  |  |  |  |  |
| N^2^-ε-dG | Precursor ion | - **^B^** | 292.10403 | - |  | [^13^C_5_^15^N_5_]-N^2^-ε-dG | Precursor ion | - **^B^** | 302.13758 | - |
|  | Product ion | 176.05704 | 176.05669 | 1.98 |  |  | Product ion | 181.07368 | 181.07346 | 1.21 |
| N^2^-P-dG | Precursor ion | 338.14599 | 338.14590 | 0.27 |  | [^13^C_10_^15^N_5_]-N^2^-P-dG | Precursor ion | 353.16518 | 353.16462 | 1.59 |
|  | Product ion | 222.09859 | 222.09855 | 0.18 |  |  | Product ion | 232.10081 | 232.10050 | 1.34 |

**^A^** Abbreviations: N^2^-ethyl-deoxyguanosine (N^2^-Et-dG); 1,N^2^-etheno-deoxyguanosine (N^2^-ε-dG); 1,N^2^-propano-deoxyguanosine (N^2^-P-dG). N^2^-Et-dG and [^13^C_2_]-N^2^-Et-dG were quantified by [^15^N_5_]-N^2^-Et-dG. N^2^-ε-dG was quantified by [^13^C_5_^15^N_5_]-N^2^-ε-dG. N^2^-P-dG was quantified by [^13^C_10_^15^N_5_]-N^2^-P-dG

**^B^** Amounts in injections of samples are typically too scarce to allow detection under full scan mode.
